# Swedish Managers’ and HR-Officers’ Experiences and Perceptions of Participating in Alcohol Prevention Skills Training: A Qualitative Study

**DOI:** 10.3389/fpsyg.2022.756343

**Published:** 2022-03-03

**Authors:** Martina Wilson Martinez, Kristina Berglund, Gunnel Hensing, Kristina Sundqvist

**Affiliations:** ^1^Department of Psychology, University of Gothenburg, Gothenburg, Sweden; ^2^School of Public Health and Community Medicine, Institute of Medicine, Sahlgrenska Academy, University of Gothenburg, Gothenburg, Sweden; ^3^Department of Psychology, Stockholm University, Stockholm, Sweden

**Keywords:** health promotion, workplace intervention, thematic analysis, alcohol policy, alcohol problem prevention, skills training experiences

## Abstract

**Objective:**

The objective of this study was to explore Swedish managers’ and HR-officers’ experiences and perceptions of skills training including a development and implementation of an alcohol policy.

**Methods:**

Semi-structured interviews were conducted with Swedish managers (*n* = 44) and HR-officers (*n* = 9) from nine different organizations whom had received skills training and an organizational policy implementation. The interviews were analyzed using thematic analyses.

**Results:**

In total, nine themes were identified as: The prevalence of alcohol problems: a wake-up call; a reminder to intervene immediately; an altered view of the responsibility of the employer; initiating conversations about alcohol: a useful toolbox; an imprecise, yet positive, memory; increased awareness of issues related to alcohol culture; I have not heard a word about a new alcohol policy; the alcohol policy: a mere piece of paper; and alcohol problem prevention: hardly a low-hanging fruit. Participants’ experiences of the skills training were positive overall.

**Conclusion:**

Various aspects of the skills training were appreciated by managers and HR-officers, including insight of prevalence statistics and employer responsibilities. Participants emphasized the value of repeated skills training occasions for retaining knowledge. Future research may investigate further in what way skills training may affect managers’ willingness to engage in workplace alcohol prevention. Since the implementation of any policy had gone unnoticed to participants, a reason for which could be related to the notion of the existing policy as “good enough” in its current condition, implementation and organizational issues, or a reluctance to address alcohol-related matters unless necessary; future research may focus on investigating in what manner alcohol policies are in fact utilized within organizations.

## Introduction

Directly attributed to 200 diseases and injury conditions and 5.9% of all deaths worldwide, harmful use of alcohol negatively impacts individual health and society (Shield et al., 2013; WHO, 2018). With 16% (19% men and 12% women) of the Swedish population (age 16–84) being classified as hazardous drinkers (The Public Health Agency of Sweden, 2020), and with a significant proportion of the population being employed (Statistics Sweden, 2017), the negative impact of hazardous and harmful drinking in Swedish organizations can be regarded as considerable (Aas et al., 2017). In an earlier study from Sweden, it has been estimated that the cost of alcohol-related absence was SEK4.3 billion for the year 2002, after subtracting reductions from beneficial health effects (Jarl et al., 2008). Especially low-quality employment in Sweden increases the risk of being diagnosed with substance use disorders (including alcohol; Jonsson et al., 2021).

Hazardous and harmful drinking are prevalent in workplaces; in particular, in sectors such as construction, entertainment, and accommodation services (Larson et al., 2007), resulting in negative consequences related to presentism and absenteeism (Ames et al., 1997; Frone and Brown, 2010; Kirkham et al., 2015; Aas et al., 2017). Prior to the outbreak of the COVID-19 pandemic, employees spent a significant proportion of their time at work which potentially optimized exposure to preventive strategies administered through workplaces. Therefore, the workplace can be regarded as a favorable arena for alcohol problem prevention. For instance, employees’ alcohol-related attitudes and drinking habits can be influenced by social norms and workplace culture (Ames et al., 2000), and detection of hazardous and harmful use of alcohol, as well as following interventions, may be facilitated through workplaces.

Various forms of workplace interventions aimed at reducing alcohol consumption among employees have been conducted; nevertheless, a meta-analysis recently concluded that the evidence for workplace interventions as an efficient way of facilitating reductions in employees’ alcohol consumption is weak (Yuvaraj et al., 2019). Significant reductions in alcohol consumption were found merely among heavy drinkers, not among employees overall. A previous meta-analysis with similar findings advocated future research with stronger methodology (Webb et al., 2009).

Notwithstanding, research suggests that the workplace may constitute a potential risk factor in terms of employee drinking, with emphasis on stress, workplace responsibility, alcohol availability, and workplace culture (Roman and Blum, 2002; Ames and Bennett, 2011). Studies have demonstrated significant relationships between alcohol availability and workplace drinking (Ames and Grube, 1999; Frone and Trinidad, 2014), indicating that physical availability of alcohol at work may constitute a risk factor for employee alcohol use. Moreover, employees’ alcohol consumption has been demonstrated to be predicted by drinking norms (Ames et al., 2000; Hodgins et al., 2009). Since drinking norms were predicted by policy enforcement (Ames et al., 2000), policy enforcement may potentially facilitate workplace alcohol problem prevention. However, to our knowledge, no recent studies have investigated the impact of policy enforcement in terms of workplace drinking.

One older study suggests that an unclear policy may constitute a risk factor in terms of workplace-related alcohol problems (Ames et al., 1992). Additionally, an RCT-study investigating the impact of policy enforcement demonstrated that the alcohol policy, while having no significant impact on employees’ alcohol consumption, resulted in an increased awareness of the policy as well as in increased employee assistance (Pidd et al., 2018). Although not necessarily facilitating reductions in alcohol consumption, enforcing organizational policies may still result in an increased inclination for managers and employees to intervene. Complementary interventions/programs that have been promising are, for example, SAMHSA,^1^ CDC Workplace health,^2^ and European network for workplace health promotion.^3^ These programs have not been applied in a Swedish context. In Sweden, the policy framework is still prevailing as the most important workplace measure.

The policy framework may also influence aforementioned risk factors, such hazardous drinking norms and alcohol availability (Ames et al., 2000). In spite of deficient evidence for its efficiency, the organizational alcohol policy remains the most applied tool associated with alcohol problem prevention in Swedish workplaces. With its existence being regulated in Swedish law insofar as organizations must have a written document concerning alcohol, the policy document should be available to all employees. Nevertheless, a Swedish study demonstrated that the policy content is frequently unknown (Tinghög, 2013).

Research investigating employees’ perceptions of the alcohol policies are limited: one Australian study demonstrated overall negative attitudes toward alcohol policies in terms of its efficiency, in particular among men and blue-collar male and female employees, and among those who typically had a higher alcohol consumption (Brown et al., 2008). One of the few studies investigating obstacles to an effective alcohol policy was a case study, its results indicating that employees found managers to be unclear regarding policy content and that alcohol availability was regarded as poorly controlled in spite of an existing policy (Ames et al., 1992). A dissertation work investigating perceptions among Swedish employees concerning alcohol problem prevention in workplaces, including employees’ views of their managers’ role in prevention, found that the responsibility of the employer was believed to be limited to severe alcohol problems, with alcohol being referred to as an individual responsibility unless causing harm to others (Tinghög, 2013). The findings demonstrated a contrasting view in relation to a public health perspective and the prevention paradox (Rose, 2001), which advocate that alcohol problem prevention proves most efficient when targeting hazardous drinkers, as well as entire organizations, in addition to merely focusing on individuals with severe alcohol problems (Butler et al., 2017). Contrastingly, the findings from the aforementioned dissertation work found that employees neither regarded the employer responsible for, nor able to, prevent alcohol problems from occurring (Tinghög, 2013), and alcohol was ought of as a very sensitive topic.

Managers’ perceptions of their own role regarding workplace alcohol problem prevention, as well as managers’ perceptions of the utility of the organizational alcohol policy, have not been investigated to our knowledge. Their perspectives may contribute with valuable knowledge for future attempts at facilitating alcohol problem prevention through workplaces, in particular, since previous attempts using workplace interventions have been inefficient (Yuvaraj et al., 2019). Managers’ roles and leadership skills have also been linked to employees’ wellbeing within other occupational health promotive domains, for instance in reducing work-related stress (Donaldson-Feilder et al., 2008), in preventing mental health issues in the workplace (Montano et al., 2017), and in the formation of safety climates (He et al., 2019). In contrast, to our knowledge, the impact of managers’ roles, skills, and leadership practices has not been investigated in relation to workplace alcohol prevention. Managers’ perceptions of important skills for enhancing detection of concurrent alcohol problems in the workplace remain unknown; as do managers’ experiences of skills-development training programs as a potential way for facilitating prevention of harmful use of alcohol. Furthermore, managers’ ideas of the utility of an organizational alcohol policy are unclear; to our knowledge, previous research has merely focused on employees’ perspectives. Managers’ experiences of organizational alcohol policy content, policy implementation, and policy enforcement may contribute with valuable knowledge in relation to in what way a policy should be constructed and utilized in order to be found applicable. Our belief is that a deeper understanding of managers’ perceptions of their roles concerning alcohol problem prevention, and perceptions in relation to an organizational alcohol policy, may contribute to facilitating future attempts at alcohol problem prevention in workplaces.

The project “Alcohol preventive interventions in working life” (KAPRI) was designed as a cluster randomized controlled trial. The aim of KAPRI was to evaluate the effectiveness of an intervention skills training including a development and implementation of an organizational alcohol policy. The effectiveness of the intervention was evaluated through measuring reductions in hazardous alcohol consumption among employees, an increase in managers’ inclination to initiate an early intervention if concerned about an employees’ alcohol consumption, as well as through improvement in managers’ organizational alcohol policy knowledge. The project was approved and carried out between 2018 and 2020, and a detailed study protocol has been published and is to be found here (Elling et al., 2020). Since there is a need for more knowledge on how managers’ experience and perceive alcohol problem prevention at work places a qualitative study was designed. The objective of this study was to explore Swedish managers’ and HR-officers’ experiences and perceptions of skills training including a development and implementation of an alcohol policy.

## Materials and Methods

### Study Design

This qualitative study explored managers’ and HR-officers’ experiences and perceptions of a skills training intervention including the development and implementation of an organizational alcohol policy. The included participants (*n* = 53) consisted of managers and HR-officers that had participated in the aforementioned KAPRI-project and had received the intervention. The primary functions of the managers were planning, organizing, leading, and controlling their organization. The HR-officers coordinated, planned, and directed the administrative functions of the organization. In total, participants from nine different organizations were included. The data collection for this study consisted of semi-structured interviews.

### Participants

All managers and HR-officers included in this study had been invited to attend two skills-development training workshops. At the initial workshop, managers and HR-officers were informed about this interview study, and those who expressed interest in participating were asked to enlist for further information by writing their name on a piece of paper. They were thereafter contacted either by telephone or e-mail by the interviewer (the first author, MW) in the order they had signed up. If a participant did not respond by phone, an e-mail was sent. An attempt was made to contact all participants at least three times per telephone and once per e-mail. Once reached either by phone or e-mail, they were asked if they wanted to participate in the study. In total, 73 managers and HR-officers had enlisted, of which 16 were unreachable and 4 declined to take part in an interview due to having changed workplace. Altogether, 53 participants were interviewed, of which 44 were managers and 9 held human resources positions. The participants included both males and females whom had various years of working experience in their current role; some less than 1 year and some more than 15 years. The majority of the participants stated having between three and 10 years of working experience in their current position. The industries of the included organizations were hospitality, construction, transportation, insurance, brewery, security, and the disability sector, with the number of employees in each organization varying between <150 and > 13,000. All organizations had at least 100 employees.

Among the managers, the most common positions were head of operations, head of department, and head of section. Other managerial positions included Chief Financial Officer, project manager, executive, and production manager. Some of the managerial positions were unspecified, with the participants referring to themselves solely as “manager.” Among the HR-officers, the majority referred to themselves as HR-specialists. For more information, see Table 1.

**Table 1:** Overview of study population n (%).

	n (%)
Managers	44 (85%; Head of operations *n* = 10, Head of department *n* = 8, Head of section *n* = 4, other managerial positions *n* = 22)
HR-officers	9 (15%; HR-specialists *n* = 4, Other human resources positions *n* = 5)
*Gender*	
Female	23 (44%)
Male	29 (56%)
*Sector*	
Hospitality	7 (13%)
Construction	9 (17%)
Transportation	4 (7.5%)
Insurance	2 (4%)
Brewery	8 (15%)
Disability	5 (9%)
Security	17 (32%)

### The Received Intervention: An Overview

The intervention that the participants had received as part of the KAPRI-project prior to being interviewed had been planned and carried out by consultants from an organization providing evidence-based prevention services for substance abuse to workplaces (Alna). Alna have arranged courses in alcohol prevention at work places the last 40 years in Sweden, in accordance with the Swedish employment law conditions. The professional title of the consultants from Alna was HR-managers. The received intervention included two components: (1) development and implementation of an organizational alcohol policy, and (2) skills-development training. In its entity, the intervention was administered by four different consultants, all of whom had been trained by the organization providing the intervention.

The development of the organizational alcohol policy implied the consultants meeting with the HR-department of each organization, in order to provide aid in revising the existing organizational alcohol policy (or create a new policy, should none exist). For more detailed information, view the study protocol (Elling et al., 2020). In total, the consultants met with the HR-department from each organization once or twice. During the initial meeting, the consultants from Alna had discussed potential revisions of the organization’s policies with the HR-department. During the follow-up meetings, which took place between 10 and 12 months after the initial meeting, the consultants from Alna followed up each organizations’ work with the policy.

The skills-development training, which was composed of two separate 3.5 h workshops, was delivered to all participants at their respective workplace. The workshop included topics such as prevention, prevalence statistics, risk factors of alcohol use disorder, warning signs indicating harmful use of alcohol, the workplace alcohol culture, responsibilities of the employer, and the concept of a conversation as a tool for intervening early. They also had practical conversational training, as well as training to find early signals of risky alcohol consumption (or other harmful use).

The focus of second workshop was to practice skills for intervening early, primarily through role-playing and case discussions, in preparation for a scenario in which a suspicion of alcohol use disorder would arise in the workplace. In addition, the second workshop focused on how to successfully develop and implement an organizational alcohol policy.

### Data Collection

**Figure 1:**
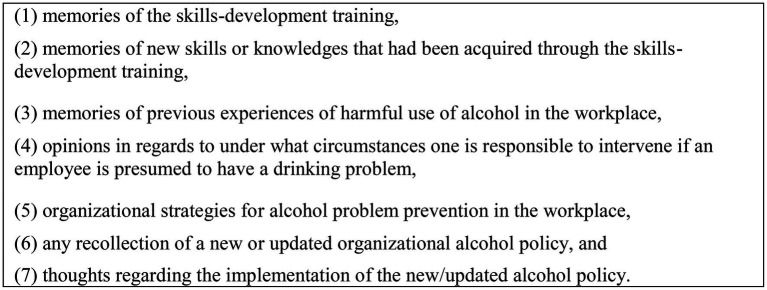
Overview of topics in the interviews.

Semi-structured interviews were conducted with 44 managers and 9 HR-officers between September 2019 and December 2019. The elapse between the intervention and the interviews were between 1–12 months. The interview questions were designed to explore participants’ perceptions and experiences of the skills-development training and of the organizational alcohol policy. The interview questions were constructed primarily by the first author (MW); a licensed psychologist with a clinical experience of working with various types of substance use disorders. All authors provided their input which resulted in a few alterations, and thereafter, the final version of the interview guide was approved by all authors. The interview guide was pilot-tested on two managers prior to being finalized, although no further revisions were made. The final version of the interview guide included a brief introduction to the interview, following questions related to the categories below (Figure 1).

The interview questions were open-ended in order to invite managers’ and HR-officers’ own thoughts and experiences; the aim was to cover their general understanding of phenomena as well as their personal experiences. Follow-up questions were asked in order to ameliorate comprehension of the respondents’ experiences and thoughts, with the number of follow-up questions varying based on how detailed the participants were in their descriptions.

The length of the interviews varied between 25 min and 90 min, although most interviews lasted for about 40 min. The interviews were conducted by telephone at a time requested by the participant; primarily, the interviews occurred during office hours. In the introduction, all participants were provided with information about procedures for ensuring confidentiality as well as being informed of the right to interrupt the interview at any time without having to provide the interviewer with an explanation. All interviews were recorded and transcribed verbatim by professional transcribers. After being transcribed, all recordings were erased. The first author (MW) interviewed all participants. The interviewer had acquired skills in interviewing, primarily due to her background as a research assistant. Moreover, recommendations of Braun and Clarke (2013) were followed in constructing the interview questions and conducting the interviews.

### Data Analysis

The interview transcripts were analyzed using inductive Thematic Analysis (TA); a method applied for identifying recurring themes, concepts, or phenomena described by participants (Braun and Clarke, 2013). In inductive TA, no attempts are made in terms of fitting data into a pre-existing framework. Initially, the first author (MWM) read through all interviews twice in order to familiarize herself with the data. Content in the interviews considered related to the research question “What were the managers’ and HR-officers’ experiences and perceptions of a skills training intervention including the development and implementation of an organizational alcohol policy?” was coded; everything else was left uncoded, in accordance with Braun and Clarke’s (2013) recommendations for selective coding. All relevant data that were interpreted as contributing to answering the research question was coded. The program used for coding was Atlas.ti. and all personal details were coded to ensure anonymity. After the coding process, each dataset was reread to ensure that nothing had been overlooked. Thereafter, the second author (KB) and the third author (KS) read the interviews. Codes and ideas were subsequently discussed between the authors prior to creating themes. The authors thoroughly followed the instructions of Braun and Clarke (2013) for TA. For instance, the idea of themes as central organizing concepts was taken into account, as were the steps for reviewing and revising candidate themes.

## Results

Nine themes were identified from the coded material. These themes reflect both experiences and perceptions of the interviewees and mirror the whole and its part to different degrees. This means that all nine themes reflect the experiences and perceptions of the alcohol problem prevention approach as designed in the KAPRI but also lift specific parts, e.g., the alcohol policy.

1. The prevalence of alcohol problems: a wake-up call
2. A reminder to intervene immediately
3. An altered view of the responsibility of the employer
4. Initiating conversations about alcohol: a useful toolbox
5. An imprecise, yet positive, memory
6. Increased awareness of issues related to alcohol culture
7. I have not heard a word about a new alcohol policy.
8. The alcohol policy: a mere piece of paper
9. Alcohol problem prevention: hardly a low-hanging fruit

### The Prevalence of Alcohol Problems: A Wake-Up Call

The first theme reflects participants’ memories of the skills-development training in terms of components that were recalled as particularly important, memorable, surprising, or unanticipated, and thus commonly referred to as a “wake-up call,” “take-home message,” or equivalent. In particular, participants exemplified the prevalence statistics of employees with a hazardous or harmful use of alcohol as an important wake-up call. Since a presupposition among participants had been that alcohol problems in workplaces were fairly uncommon, this newly acquired knowledge was referred to as particularly memorable.

*“What you take with you from this kind of training is that you have a bit of an ‘ah-ha’ experience, that alcohol problems are more common than you might think.”*

The aforementioned prevalence statistics were also considered important by participants due to it resulting in increased awareness of the fact that there were presumably undetected alcohol problems in the workplace.

*“Yeah, but the fact of the matter is there are actually a lot of hidden alcohol problems that you do not see.”*

Participants’ reflections regarding what specific knowledge constituted a wake-up call were often associated with their own previously held misconceptions that were altered during the skills-development training. For instance, an often referred to wake-up call included knowledge about the prevalence of individuals with a hazardous use of alcohol. Before the skills-development training, participants expressed having held the misconception that only heavy drinking, and not hazardous drinking, gave rise to health- and work-related problems. Since any problems associated with hazardous drinking had previously been unbeknownst to participants, increased knowledge about its prevalence and consequences were referred to as particularly important and memorable.

*“I used to think that only very few people succumbed—but if it happened, it was severe, it was obvious—but that there were so many people who engaged in risky behaviour.”*

In addition, participants referred the skills-development training making them realize that they themselves might be consuming too much alcohol; often described as a result of the newly acquired knowledge in terms of what constitutes hazardous alcohol consumption. A common presupposition brought up by participants was that one could consume a rather high amount of alcohol on a weekly basis without simultaneously engaging in a hazardous drinking behavior; this presumption was described by participants as having altered. Therefore, participants referred to increased knowledge of hazardous drinking as a wake-up call in terms of their personal health and lifestyle.

*I guess that was what struck me most—how little alcohol you need to consume per week to reach the level of a health hazard.*

In addition, the skills-development training was referred to as a wake-up call in terms of raising awareness of concurrent alcohol problems in the workplace. Being presented with a list of warning signals indicating an alcohol problem, participants explained being confronted with the realization that employees under their responsibility were displaying relevant warning signals. There were participants disclosing that the warning signals presented in the skills-development training aided them in discovering an employees’ alcohol problem.

*Before taking this training I went around thinking there was something weird going on here. We have a history here that includes both stress and short-term absences. And after this training, I can clearly see what I, as a manager, have not wanted to see.*

A final notion among participants was that the skills-development training resulted in their becoming more reflective and open-minded, due to questioning prior beliefs about alcoholism. Participants exemplified having prejudices related to alcohol that had been altered, and some referred to their thinking as less black-and-white concerning problem drinking. Prior to the skills-development training, participants referred to having categorized individuals as either a problem drinker or a normal drinker, with very little in between. The increased awareness of the consequences, as well as prevalence, related to hazardous drinking was referred to as having resulted in a more nuanced notion of what constitutes problematic use of alcohol.

### A Reminder to Intervene Immediately

The second theme reflects perceptions of managers and HR-officers whom expressed having acquired knowledge in the field prior to their attending the skills-development training sessions. A notion was having learnt new things in spite of having attended similar skills training programs in the past.

*This is not the first time I’ve listened to this kind of thing. I thought they actually took some new approaches to what is important, what you should be looking for*.

Other participants were referring to already having acquired knowledge based on previous experience, for instance in regards to warning signals indicating an existing alcohol problem in the workplace. Nevertheless, in regards to the perceived utility of the two skills-development sessions, the notion was that repetition was important in order to retain previously acquired skills.

*I’ve actually heard that before, too, but these are things you need to be reminded of now and then, I think.*

Furthermore, participants emphasized the importance of being reminded to intervene immediately should a suspicion arise. Moreover, being reminded of the existence of alcohol problems overall was considered important; as was the reminder to initiate conversations with concerned employees as soon as there were signals indicating an alcohol problem. A shared notion was that it was generally easier to overlook harmful use of alcohol than intervening, in hope of that someone else in the workplace would handle the situation.

*Well, you know, I’ve been doing this for so long, but I’ve learned that it’s good to be reminded that you need to react promptly. That you cannot just ignore the problem and hope it’ll resolve itself or that someone else will deal with it.*

Moreover, participants described the skills-development training as a form of encouragement to intervene in future situation; in particular, when in doubt about how to proceed. Another held notion was that one needs to attend recurring skills-development programs regarding alcohol in the workplace, in order to stay fully informed.

*I think you always get something out of this kind of training—you’ll never know all there is to know.*

### An Altered View of the Responsibility of the Employer

The third theme reflects notions in regards to what extent the employer is considered responsible for handling an existing alcohol problem in the workplace; a view that was described by participants as having altered due to the skills-development training. For instance, participants recalled that the responsibility of the employer had been clarified to them. Moreover, some participants mentioned not being informed at all in terms of the employer’s obligations prior to the two skills-development sessions.

*I have to admit I wasn’t really aware of the employer’s responsibility in relation to these issues. After taking the training, I see that we should perhaps have acted differently.*

A notion was not having been familiar with the responsibility of the employer regulated in Swedish law, in particular in terms of obligations related to an employee’s rehabilitation. For instance, managers declared having previously believed that the individual had a more pronounced responsibility; a view that was described as having shifted as a consequence of the skills-development training. Having underestimated the lengths to which the employer was considered responsible for rehabilitation in a scenario when an employee was diagnosed with an alcohol problem was also exemplified.

*Earlier, I guess I thought it was the responsibility of the individual rather than the workplace, and I think I’ve changed my outlook a little, regarding responsibility, after taking this training.*

Although the skills-development training was considered clarifying in some respects, participants still expressed hesitation in regards to the responsibility of a mangers or employer to detect harmful use of alcohol, as well as under what circumstances a manger is obliged to intervene if suspecting, or becoming aware of, an alcohol problem. A described notion was that the responsibility was unclear in various circumstances, especially in terms of the employer’s responsibility to prevent harmful use of alcohol, as well as dealing with hazardous alcohol consumption. Participants also pointed out that there was a fine line between work and private life, which complicated the responsibility to intervene in situations when an employees’ work performance had not been palpably deteriorated. A notion was that it was debatable whether an employer should be responsible for alcohol problems not affecting job performance; participants’ notions varied in this respect with some participants considering the employers’ responsibilities as pronounced, whereas others expressed further skepticism in terms of the employer’s responsibilities. Some participants expressed concern in terms of going too far as a manager or employer. Such concerns included whether accidentally accusing an employee of having a problem could result in losing one’s job or being sued by the employee.

*If I’m going to have a conversation with someone I suspect may have alcohol problems, it’s always there in the back of my mind, thinking about what to do and say. Because that person might have no problem at all, and you could then possibly lose your job*.

Ultimately, participants expressed the importance of raising employee’s awareness of the responsibility of the employer; their assumption being that most employees were not sufficiently informed in the matter. A notion among HR-officials was that knowledge of the responsibility of the employer could be deficient among managers and employers as well, and in particular to new managers; something that was regarded as problematic.

*It’s the employer who has the responsibility and the obligation—that’s extremely important. I think many companies do not really know what responsibilities they have*.

### Initiating a Conversation About Alcohol: A Useful Toolbox

The fifth theme reflects the managers’ and HR-officer’s notions of skills and knowledge acquired as a result of the skills-development training sessions. For instance, participants referred to the information provided as constituting a useful toolbox, which could come in handy in the future.

*I had the impression that all of the participants went away with helpful new tools for their toolbox.*

**Figure 2:**
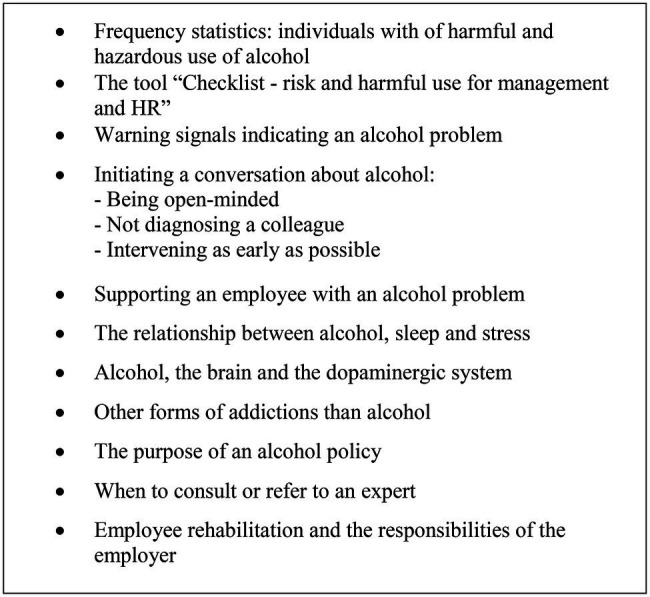
Overview of tools and knowledge acquired during the skills-development training.

When asked further about what specific tools and knowledge had been acquired, the below-described examples were referred to by participants (Figure 2).

Participants often referred to having learnt about the necessity of having an open-minded approach, being non-judgmental and not diagnosing an employee as a result of the skills-development training.

*The first thing I learned was that I, or the boss and I together, must refrain from diagnosing the employee.*

Participants also expressed that the skills-development training has helped them re-filling their knowledge base in regard to identifying early warning signals indicating an alcohol problem. A notion was that signs of alcohol problems are easily overlooked unless one had sufficient knowledge.

*I mean, I think it’s useful for everyone to understand the point about early signs. I do not think we notice them particularly often.*

Participants recurringly referred to a tool “Checklist - risk and harmful use for management and HR” that had been introduced (see attachment x) during the first session; a document which was described as potentially useful for detecting warning signals as well as constituting a guide for initiating a conversation with an employee about alcohol. Participants expressed appreciation for having been provided with the checklist on a USB-stick by the consultants that way the tool could be utilized in the future if needed.

In addition, participants regarded the role-playing included in the skills-development training as interesting and useful; an exercise that implied practicing imitating a conversation about alcohol with collogue about his or her alcohol consumption. These conversations were referred to as awkward and sensitive and thus considered difficult to initiate. A notion was that the role-playing exercise was useful in terms of daring to initiate a conversation at all, in particular in a situation in which there is any uncertainty in regards to whether an employee has an alcohol problem.

*I think the primary reason why we fail to address it is that it’s sensitive*.

Participants also referred to the role-playing as being clarifying in regards to how difficult it is to ask an employee about alcohol; some expressed not having pondered much upon prior to the exercise. Ultimately, participants believed that the skills-development training had increased their inclination to initiate conversations about alcohol with employees, since the role-plating exercise resulted in them feeling more comfortable in taking action.

*Addressing these problems seems a little easier now, I must say. It feels like we have done it once, and we can do it again.*

Nevertheless, when asked to what extent the aforementioned acquired tools had been considered useful in practice, few participants recalled actually having initiated a conversation with an employee about alcohol since the skills-development training took place.

### An Imprecise, Yet Positive, and Memory

The fifth theme reflects participants’ overall memory of the skills-development training; a memory that was described as positive and vague. Common words used by managers and HR-officers for describing their memory of the two training sessions included “interesting,” “good,” “very good,” “positive,” “important,” and “appreciated.”

*I remember feeling that the training was a positive experience.*

While being described as positive and interesting, few specific details of the skills-development training sessions were recalled by participants. Although topics that had been found interesting were referred to, such as aforementioned warning signals indicating alcohol use, the referred to concept and phenomena were described in a general manner, typically devoid of details. For instance, managers referred to a mere memory of having learnt new things, rather than what specific tools and knowledge had been acquired.

*I remember we talked about this difficult conversation and how it has to be initiated by us, but honestly, I do not remember that much of it.*

Common phrases used for describing the overall experience of the two sessions included “I cannot remember,” “it was too long ago to remember,” and “you should have asked me one year ago.” Participants related their forgetfulness either to the fact that too long a time had passed since they attended the skills-development training or that no situation had occurred in the workplace that had enabled them to utilize the acquired skills in practice.

*That was about a year ago, so to be quite honest I have not had to deal with the kinds of things I learned about. But I remember I thought it was a good training.*

### Increased Awareness of Issues Related to Alcohol Culture

The sixth theme reflects participants’ notions of the skills-development training shedding light on obscure issues related to their organizations’ alcohol culture. Participants mentioned the two sessions resulting in their becoming increasingly aware of issues concerning rules, norms, and alcohol culture within their workplace; some of which were considered unhealthy and had not previously been regarded as problematic. A notion was that, since rules, norms and alcohol culture were seldomly discussed within the organization on a regular basis, potential issues had remained obscure.

*These issues do not pop up every day—that you are discussing policy or procedures or dilemmas and so on.*

Participants exemplified their celebrating with alcoholic beverages during office hours, and their arranging after work with alcoholic beverages at the office, as something not previously regarded as problematic. At the skills-development training, it became clear to participants that the above-mentioned norms were not merely potentially hazardous; they additionally violated their organizations’ alcohol policy. Participants shared the notion that the consultants had been skilled at pinpointing discrepancies between their organization’s policy, norms, and alcohol-related behavior.

In addition, participants brought up feeling appreciative that external consultants had provided their organization with the skills-development training program, instead of someone from within their own organization. A belief among participants was that external consultants had managed to shed light on things that were considered difficult to discuss within the workplace. Some participants related the presumed difficulty to talk about alcohol-related topics to the belief that alcohol was considered a sensitive subject.

*Lectures by external researchers are interesting, because they are a whole different thing from just sitting around and discussing something with your own group.*

Moreover, managers and HR-officers expressed their becoming increasingly aware of the organization’s alcohol culture overall due to the skills-development training. A notion was that novel within-organization discussions about alcohol had evoked, for instance in relation to upcoming corporal events such as Christmas Table. Alcohol as an increasingly emerging topic was referred to as long-term consequence of the skills-development training by participants.

*It’s a change—we are focusing on it more, talking about it more now than we did two years ago, definitely. And this training is part of that, you know. And that we have done it. Afterwards, we have definitely started talking about it much more in various situations.*

### I Have Not Heard a Word About a New Alcohol Policy

The seventh theme constitutes reflections among managers and HR-officers regarding the development and implementation of an organizational alcohol policy, which had been part of the received intervention. In the intervention, the organizations were to encouraged to either revise their existing policy or create a new alcohol policy. Thereafter, the revised policy was planned to be implemented in each organization, in accordance with the planned intervention.

In this theme, there was a distinguishable discrepancy between managers’ and HR-officers’ notions in terms of the familiarity of the policy. The interviewed HR-officers, some of which had been part of the group meeting with the consultants concerning the policy, referred to having knowledge of a plan to revise the policy. In contrast, few of the interviewed managers mentioned having heard of any intention to revise the organizational alcohol policy; let alone that updating the policy had been part of the received intervention.

*I have not heard a single word about it. So I do not imagine we have any express strategy or that there is any plan for it in place at the moment.*

A notion among managers was already having access to an existing, fully functioning policy, the content of which they were familiar with. Another notion among managers was sheer bewilderment in regards to why on earth the policy should be revised when the existing one was about as restrictive as it could be.

*There have not been any revolutionary changes in any policy. But rather, it’s quite… yes, like before, that we have zero tolerance. What more, beyond zero tolerance, can you have?*

A reflection among managers was their ignorance regarding the new policy was due to their not being involved in the policy revisions, and the managers often referred to the HR-department as being primarily responsible for revising the policy. An additional reflection was having been briefly informed about a potentially new policy during the skills-development training sessions, while simultaneously stating not having heard of it since. Even when the existing policy was referred to as old and in need of updating, often still no change had occurred according to the managers’ beliefs.

*I know we have had an alcohol policy before, which was pretty old and out-of-date and that we were going to update—that much I know. But I have not seen any sign of that.*

Managers referred to the size of their organization as a potential reason for their lacking insight; a revision of the policy could have easily gone unnoticed to them according to their notion. Notions among HR-officers, many of whom had been involved in the group assigned to revise the policy, differed from mangers’. HR-officers rather referred to an intention to revise the policy, while simultaneously declaring that alternations had not been finalized. A notion among the HR-officers’ was that more urgent matters had evoked during the process that had required immediate attention, inevitably postponing the revision of the policy.

*Unfortunately, I have prioritized other things. And I’ve felt that, well, we have the old one, and it works. But the new one will also be good, when it arrives, but I’ve actually put it on the back burner.*

HR-officers also referred to having scheduled a future meeting assigned to the policy, during which the revisions of the policy would resume. Neither managers nor HR-officers accounted for any strategic plan in terms of creating or implementing a new organizational alcohol policy. Overall, participants expressed doubt in regards to whether the policy had in fact been implemented, let alone accepted by the employees; even under circumstances when a new alcohol policy had been created and all employees had been enforced to sign it.

*Given that we have put our signatures to our agreement that we’ll be fired if we contravene the policy, we have all been obliged to accept it. But I do not think everyone is happy about it and bought in to it, so to speak, with their heart and soul.*

### The Alcohol Policy: A Mere Piece of Paper

The eighth theme reflects managers and HR-officers’ opinions of the utility of an alcohol policy. A recurring notion was the policy being regarded as a piece of paper rarely read, rather than considered a tool in terms of alcohol problem prevention in the workplace.

*I would say the policy is actually only a product on paper—so for sure you can have loads of policies and say ‘Look, we have policies,’ but if nobody cares about them, they are not helping in any way.*

A belief among participants was that managers in leading positions had the ability to increase employees’ awareness of alcohol-related matters, as well as affecting employees’ attitudes, more efficiently than the mere document that constitutes an organizational alcohol policy. It was exemplified that a leading manager openly expressing interest in alcohol problem prevention and acting responsibly in front of the employees could potentially influence employees’ alcohol-related attitudes and behavior for more efficiently than a mere paper policy. Moreover, participants referred to previous experiences of having a manager whom in their opinion had influenced employees’ behavior in terms of alcohol.

*A focused effort, by some manager or some particular function, to make personnel aware of the issue—highlighting it and getting people to talk about it—not just today, but keeping the issue alive for a time. That, I think, would have more effect than the existence of a written policy.*

Participants’ notion was that the alcohol policy itself constituted a useful tool if uncertain about something relating to alcohol, for instance if suspecting an alcohol problem in the workplace. Moreover, the policy was regarded by participants as a useful tool in a situation in which an employee would misbehave; in that potential scenario, the participants expressed feeling confident in being able to refer to the rules written in the policy. Nevertheless, managers pointed out that they would rather consult the HR-department than reading the policy if a difficult situation occurred. Another notion was doubtfulness in terms of successfully keeping the topic of “alcohol” alive merely through a policy; notions were that there were more efficient ways of engaging in alcohol problem prevention than through a policy. In addition, participants expressed some reasons for doubting the utility of a policy: for instance, participants referred to the policy being unknown and hence inaccessible to a considerable proportion of the employees.

*It has to be extremely easily accessible, I think. Plus, that an effort is made to find occasions to bring up the subject and get people talking about it. Because I do not think waiting for natural occasions to arise is going to get us anywhere.*

In order to retain recurring discussions of alcohol and the workplace, participants referred to the importance of finding occasions for discussing alcohol problem prevention within the organization. At the same time, it was considered difficult to find occasions for bringing up alcohol “out of the blue,” since alcohol consumption was either regarded a sensitive subject or a private matter. Rather, participants believed that the topic of alcohol was regularly brought up due to a negative incident having occurred. However, some participants had the belief that the more frequently alcohol was discussed, the less sensitive the topic became overall.

*The more we talk about it, the less sensitive it is.*

Albeit the notion that few employees were in fact aware of the content of the organizational alcohol policy, participants still referred to potential strategies for increasing employees’ policy awareness. For instance, methods such as sending out the policy along with the salary specification, enforcing all employees sign the policy document, or attaching the paper version of the policy on office walls where all employees would inevitably spot it, were exemplified as strategies for increasing employees’ policy awareness.

*It was mailed out together with the monthly salary statements. So the policy was sent to their homes where they could read through it at their leisure.*

A final notion in regards to the policy was that an already vast number of policies within the organizations resulted in a general caution to spread new policies overall. Some managers referred to something alike a policy aversion or an overall tiredness of reading policies.

*We have so extremely many policies and it’s so easy for us to become… we are careful about disseminating any policies.*

### Alcohol Problem Prevention: Hardly a Low-Hanging Fruit

The final theme reflects the participants’ general notion of the concept of alcohol problem prevention; a topic that was frequently addressed by participants while recalling the received intervention. While holding the belief that alcohol problem prevention is important, it was simultaneously referred to as a difficult task for workplaces. Obstacles mentioned included lack of time, deficient resources, deficient interest, changes within the structure of organizations, and employee turnover. Another held notion was that alcohol problem prevention was seemingly not prioritized within their organization unless an alcohol problem becomes noticeable in the workplace. Since there are constantly pressing issues to deal with that requires immediate attention, alcohol problem prevention was believed by participants to be deprioritized as a consequence.

*It’s a question of priorities. By ‘alcohol abuse’—I mean, it gets put higher on the priority list if something happens.*

Additionally, a notion among participants was that alcohol problem prevention was thought of as a long-term investment by their employers. Simultaneously, participants held the belief that long-term goals were often overlooked by employers and that investments resulting in more immediate rewards were prioritized over alcohol problem prevention.

*I think it’s a matter of prioritization. And an alcohol policy is a long-term investment. And in our day-to-day work, if we do not get clear direction, I think we end up paying attention to the low-hanging fruit… it’s easier to deal with the low-hanging fruit, which has a short-term effect.*

An additional belief among the participants was that alcohol problem prevention would not be prioritized since it required resources while at the same time not considered palpably lucrative. Participants held the belief of alcohol problem prevention as unjustified economically, since it was thought of as time-consuming yet difficult to evaluate from an economic viewpoint. An additional belief was that increased awareness of the economic losses directly attributed to alcohol consumption could potentially increase managers’ and employers’ interest in alcohol problem prevention.

*You have to underline the cost of ill-health to the workplace and the cost of ill-health related to alcohol abuse.*

Moreover, a notion was that alcohol problem prevention was deprioritized due to alcohol use being considered an individual responsibility rather than primarily the responsibility of the employer.

*They do not think it’s cost effective to spend time talking about these things, because again, the thinking is that it’s the responsibility of the individuals themselves to deal with the problem*.

Besides the economic viewpoint, a notion was that attributing alcohol problem prevention to organizational values and branding could potentially increase managers’ inclination to prioritize alcohol problem prevention, although participants referred to this viewpoint as speculative rather than based on personal experience.

*It’s like the second-strongest motivator, after the economic aspect. That you have a clear foundation in terms of values, and that you can benefit from such clarity.*

## Discussion

To our knowledge, this qualitative study is the first to explore managers’ and HR-officers’ experiences and perceptions of a skills training intervention directed at prevention of alcohol problem. Few studies have been directed at managers and HR-officers in spite of their key positions and roles in promoting work place based actions to prevent alcohol problems. The added value of exploring these experiences and perceptions is a better adaptation of future interventions at work and of guidelines and recommendations from authorities with responsibility of work environment and workers’ health.

We identified nine themes. “The prevalence of alcohol problems: a wake-up call” (1) reflects the participants’ notion of the skills-development training providing them with important knowledge previously unbeknownst to them; the frequency statistics of hazardous and harmful drinking being exemplified as such. “A reminder to intervene immediately” (2) conveys the perceived importance of being reminded not to hesitate to intervene should a suspicion of alcohol problems arise; this was particularly emphasized by participants with previous knowledge in the field. “An altered view of the responsibility of the employer” (3) describes the notions of having ameliorated one’s understanding of the employer’s responsibilities in a situation involving alcohol problems. “Initiating conversations about alcohol: a useful toolbox” (4) reflects the notion of having acquired new knowledge and tools that could be used for initiating conversations about alcohol with employees. “An imprecise, yet positive, memory” (5) reflects participants’ recollection of the skills-development training as positive overall, even though few specific details were recalled. “An increased awareness of issues related to alcohol culture” (6) conveys issues concerning organizations’ alcohol cultures that became apparent due to fruitful discussions that emerged as a result of the skills-development training. “I have not heard a word about a new alcohol policy” (7) reflects the notion of a presumed non-existing new or revised alcohol policy, alternatively, that any information concerning a new or revised policy had gone unnoticed to the participants. “The alcohol policy: a mere piece of paper” (8) reflects participants’ ideas of the utility of an organizational alcohol policy, with the policy often referred to as a rarely read paper product. The final theme “Alcohol problem prevention: hardly a low-hanging fruit” (9) reflects participants’ referral to workplace alcohol problem prevention as a low priority matter, due to it being considered unjustifiable from an economic viewpoint.

The findings from this study indicate that a skills-development training can be found informative and instructive by participants, albeit the concurrent notion of the skills-development training as a mere positive memory devoid of specific details also existed. This might reflect that the training was too short since research has shown that repetition is important in all learning situations. However, the possibility to extend the skills training is hampered by an intense and time constrained working life. Future interventions could test digital solutions to facilitate reinforcement.

Of specific importance are the findings that the participants lacked insight concerning the organizational alcohol policy. This is important given that work place alcohol policies are the cornerstone of Swedish work place based alcohol problem prevention. Participants described it as a paper product rather than an active and changing frame for dialog and action. Obstacles that constitute some of the common difficulties in relation to implementations, such as good communication and information systems (Al-Ghamdi, 1998), were exemplified by participants in relation to their lacking insight concerning the alcohol policy. In addition to the aforementioned obstacles, participants emphasized difficulties locating policy documents, too vast an organization to know about everything going on, as well as too many already-existing policy documents resulting in something alike a “policy aversion.” It seems as if there is room for both practice changes and future studies on how an alcohol policy could be better adapted and communicated to support managers in their responsibilities to prevent alcohol problems, keep a safe work place, and protect workers’ health.

A notion among participants was that an already functional policy was in place, which was commonly referred to as “good enough.” The existing policy version still appeared relevant to the interviewed participants and a shared assumption was that it was neither urgent nor necessary to revise the existing policy. The idea of the policy as “good enough” may be interpreted in the light of previous research which demonstrated that employees think of alcohol as a very sensitive topic when discussed within the workplace (Tinghög, 2013); a finding also identified in this study. In addition, this study identified a perceived difficulty in terms of bringing up alcohol “out of the blue”; rather, participants preferred to have a valid reason for addressing the topic of alcohol, such as an alcohol-related incident having occurred in the workplace. Since alcohol was perceived as an uncomfortable topic, the contentedness with the existing policy could potentially be related to an emotional avoidance in terms of dealing with sensitive alcohol-related matters. Similarly, willful blindness or ignorance concerning any potential improvements of the existing policy may constitute a similar avoidance in terms of addressing the sensitive alcohol-related topics in the workplace. Interestingly, participants pointed out that the topic of alcohol became less sensitive if frequently brought up in the workplace. This perception can be related to the concept of habituation that repeated applications of a certain stimulus may result in a decreased emotional response (Thompson, 2009). A policy development based on these findings could be to develop the Swedish Work Environment Act and associated prescripts to be more specific concerning alcohol issues to be part of the systematic work with the work environment. This could make it easier for managers to actually bring up alcohol issues in spite of its sensitivity and, in some cases, against prevailing norms at work places with an allowing attitude to alcohol.

An additional explanation to the participants’ perceived contentedness with the policy may be related to their coinciding notion of alcohol problem prevention being a low priority matter in workplaces. Deficient incentive and insufficient time were brought up by participants as potential obstacles for alcohol problem prevention as well as for policy implementation. The notion of alcohol problem prevention as unjustifiable from an economic viewpoint in comparison with endeavors generating more obvious and immediate rewards, regarded from an employer’s perspective, could constitute an additional explanation for the notion of the policy document as “good enough” in its current version.

In this study, the alcohol policy was not emphasized as an important tool in preventing harmful and hazardous use of alcohol, in accordance with previous findings (Brown et al., 2008; Tinghög, 2013), and the perceived utility of an organizational alcohol policy was limited to situations in which one was uncertain about how to proceed. Beyond this purpose, the policy was referred to as a mere document in which to look for aid should an incident occur. Any notion of a policy as a useful tool, read on a regular basis, referred to and utilized for preventive purposes, did not appear to exist among the interviewed participants. Perceptions of the policy as constituting little more than a mere document may have been linked to the participants’ lacking insight in terms of a new or revised organizational policy, as well as their disinterest in ameliorating the current version of the policy.

The participants’ perceptions of the organizational alcohol policy may additionally be linked to notions of the responsibility of the employer regulated in Swedish law; the participants perceived the employer’s responsibilities as clear in terms of rehabilitation, albeit palpable hesitation was expressed in relation to responsibilities in preventing and detecting harmful use of alcohol, and under what circumstances a manager should be obliged to intervene if suspecting an alcohol problem. In accordance with an injunction in the Swedish constitution (The Swedish Work Environment Authority, 1994), an employer is responsible for rehabilitation should an employee be diagnosed with alcohol use disorder. Moreover, the employer is declared responsible for clarifying rules if an employee is under the influence of alcohol or other substances at work (The Swedish Work Environment Authority, 1994); rules that should “preferably” exist in written form (i.e., through an alcohol policy). However, it was unclear to participants to what extent the employer should be responsible for alcohol-related ill-health existing among employees; an uncertainty that may be traced to Swedish law insofar as the extent to which alcohol should be interpreted as ill-health is not specifically stated beyond alcohol use disorder. Uncertainty in regards to what constitutes alcohol-related ill-health, in combination with perceptions that alcohol is a sensitive and private matter (Tinghög, 2013), may have prevent policy enforcement as well as the unwillingness to engage in alcohol problem prevention as an employer or manager, unless an urgent situation occurs.

Beyond the policy, previous research has emphasized managers’ leadership styles as important in maintaining a safe workplace (Kapp, 2012; He et al., 2019), and in promoting employees’ health (Donaldson-Feilder et al., 2008; Montano et al., 2017). In accordance with previous findings, participants in this study regarded managers in key positions leading by example as having a more important role in facilitating alcohol problem prevention and promoting healthy drinking habits, than enforcing a policy document. Thus, we recommend that future research investigates managerial leadership further in relation to workplace alcohol problem prevention.

The results from this study indicate that managers appreciated learning more about harmful and hazardous alcohol consumption as well as tools which could be utilized to approach employees with a potential alcohol problem. Moreover, prevalence statistics was referred to as unbeknownst to many participants prior to the training and thus emphasized as important. Since the findings from this study indicate that skills training was appreciated by managers and the managerial role was considered relevant in relation to workplace alcohol problem prevention, we suggest that future research focuses on ameliorating understanding of these aspects. For instance, the extent to certain aspects of skills training can be interpreted as useful for detecting alcohol problem in workplaces, as well as for facilitating initiation of an intervention, could be investigated further using quantitative methods. Moreover, the extent to which skills training may increase engagement and interest in workplace alcohol problem prevention overall remains unknown.

One limitation of the study was that we could not guarantee that the telephone interviews were confidential. Since our study interviewed a non-random sample of participants, all of whom expressed interest in being interviewed, the transferability of the results may have been affected. For instance, opinions among participants who voluntarily participate in an interview, as in this study, may differ from opinions of managers and HR-officers overall. Therefore, we recommend that future research investigates to what extent the findings from this study may be generalized, using quantitative methods. Moreover, future research could continue investigating what knowledge and tools are perceived useful by managers in facilitating alcohol problem prevention, besides the alcohol policy document.

Since this study based its findings on an intervention administered as part of an RCT-study, the findings from this study were inevitably affected by the manner in which the intervention was conducted. For instance, ignorance among managers concerning a new organizational alcohol policy could potentially be explained by the methodology utilized in RCT and problems related to administering the intervention, rather than through managers’ general perceptions of an alcohol policy. Moreover, the skills-development training was administered by different consultants; thus, the manner in which the information was presented to the participants may have affected notions of the utility of the skills-development training as whole, as well of the utility of specific information; hence the overall findings of this study.

## Conclusion

The findings from this study indicate that various aspects of the skills training were appreciated by managers and HR-officers, including insight of prevalence statistics and employer responsibilities. Participants emphasized the value of repeated skills training occasions for retaining knowledge. Any implementation of an alcohol policy had seemingly gone unnoticed to the participants; a reason for which could be related to the notion of the existing policy as “good enough” in its current condition, implementation and organizational issues, or a reluctance to address alcohol-related matters unless necessary. The findings can also be used in policy development, e.g., specify alcohol issues as part of managers’ work environment responsibilities and as basis for public health communication regarding alcohol at work. We suggest that future research investigates further in what way skills training may affect managers’ willingness to engage in workplace alcohol prevention as well as in what manner, and to what extent, alcohol policies are in fact utilized within organizations.

## Data Availability Statement

The original contributions presented in the study are included in the article/supplementary material, further inquiries can be directed to the corresponding author.

## Ethics Statement

The studies involving human participants were reviewed and approved by The Ethical Review Board of Stockholm Region (dnr 2018/634–31/5). The patients/participants provided their written informed consent to participate in this study.

## Author’s Note

This article is part of MM’s doctoral thesis.

## Author Contributions

MM coordinated the study, interviewed all participants, coded the data, and wrote the first draft of the manuscript. All authors contributed to analyzing the data and choosing themes and contributed to revising the first drafts and finalizing the manuscript.

## Funding

This project was funded by The Public Health Agency of Sweden (dnr: 027812017, 033332018, and 038432019).

## Conflict of Interest

The authors declare that the research was conducted in the absence of any commercial or financial relationships that could be construed as a potential conflict of interest.

## Publisher’s Note

All claims expressed in this article are solely those of the authors and do not necessarily represent those of their affiliated organizations, or those of the publisher, the editors and the reviewers. Any product that may be evaluated in this article, or claim that may be made by its manufacturer, is not guaranteed or endorsed by the publisher.

## Footnotes

^1^ https://www.samhsa.gov/workplace/resources

^2^ https://www.cdc.gov/workplacehealthpromotion/index.html

^3^ https://www.enwhp.org/?i=portal.en.workplace-health-promotion
